# Metagenomics Study of Contaminated Sediments from the Yamuna River at Kalindi Kunj, Delhi, India

**DOI:** 10.1128/genomeA.01379-17

**Published:** 2018-01-04

**Authors:** Nishi Sahu, Maushumi Deori, Ilora Ghosh

**Affiliations:** a School of Environmental Sciences, Jawaharlal Nehru University, New Delhi, India

## Abstract

The Yamuna River is the backbone of domestic, irrigation, and industrial activities in Delhi, India, yet the complex dynamics of its microbes and their contribution to biogeochemical cycles in a polluted environment remain elusive. This is an introductory report describing the microbial community in the Yamuna River, using high-throughput metagenomics.

## GENOME ANNOUNCEMENT

Originating in the Yamunotri Glacier in the lower Himalayas, the 1,376-km-long Yamuna River drains 366,223 km^2^ of the North Indian basin (1). Widely referred to as the lifeline of Delhi, India, the Yamuna waters are extensively harnessed for various household, industrial, and irrigation purposes in the capital (2). In Delhi, its 46-km stretch receives wastes from 18 drains, which contribute to 70% of its total pollution load (3). While the central monitoring authority, the Central Pollution Control Board (CPCB), cites untreated sewage and industrial wastes as the primary causes of concern, diffused sources of pollution, like agricultural pollution, cattle washing, cloth washing, open defecation, and domestic wastes are known to aid in the proliferation of pathogens (4). As the river moves from north to south in Delhi, CPCB reports high levels of biological oxygen demand (BOD) and fecal coliform and extremely low levels of dissolved oxygen (DO). Chemically intensive fiscally exhaustive methods to contain the pollution through effluent treatment plants, etc., have yielded no promising outcomes. Current studies attest to the fact that the present deplorable condition of Yamuna is beyond any physical or chemical redemption, and further restoration projects should focus on strategies that exploit the internal mechanism of microbes for the degradation of pollutants (5, 6). The present study overcomes the limitations of the culture approach by applying a culture-independent approach, like metagenomics, for characterization of the microbial community (7, 8).

The river sediments were collected from a suburban industrial site at the Okhla barrage (28.5457°N, 77.3105°E) located on the banks of the Yamuna River in March 2017 and stored at 4°C until further analysis. The DNA was extracted using a PowerMax soil DNA isolation kit (Mo Bio Laboratories, Inc., Carlsbad, CA, USA) and sequenced using an Illumina platform. The amplicon library (2 × 300 MiSeq) was prepared using Nextera XT index kit (Illumina, Inc.). Sequencing was done using MiSeq system. The software QIIME (9) has been used to obtain microbial operational taxonomic units (OTUs) and their abundances in each of the samples.

A significant percent abundance (>15%) at the phylum level was shared by *Proteobacteria* (33.68%), *Bacteroidetes* (24.07%), and *Firmicutes* (19.06%). A diverse microbial community exists, with their percent abundance (<10%), as follows: *Synergistetes*, 8.15%; *Verrucomicrobia*, 4.05%; *Cyanobacteria*, 2.41%; *Actinobacteria*, 1.92%; *Fusobacteria*, 1.66%; *Planctomycetes*, 1.21%; *Chloroflexi*, 0.89%; *Spirochaetes*, 0.73%; *TM7*, 0.55%; *Tenericutes*, 0.47%; *Acidobacteria*, 0.39%; *Lentisphaerae*, 0.3%; *Euryarchaeota*, 0.09%; *NKB19*, 0.04%; *OP8*, 0.03%; and *BRC1*, 0.03%. At the order level, *Bacteroidales* (23.09%) were found to be the most abundant. This diverse abundance represents the complex mechanism occurring inside the ecosystem.

The metagenomics analysis is consistent with pollution study reports of the rapidly declining health of the river; the higher content of anaerobic microbes in the study suggests unchecked pollution is progressively creating the anoxic conditions of the river within the city. The report also provides crucial insights into individual microbial population dynamics in response to high pollution and contributes valuable information to ongoing remediation studies on the Yamuna River.

### Accession number(s).

Our metagenome sequence has been uploaded on the NCBI website under the accession number SRX3209816.
